# Generating 3D-cultured organoids for pre-clinical modeling and treatment of degenerative joint disease

**DOI:** 10.1038/s41392-021-00675-4

**Published:** 2021-11-12

**Authors:** Ye Sun, Qiang Wu, Kerong Dai, Yongqing You, Wenbo Jiang

**Affiliations:** 1 Department of Orthopaedics, The First Affiliated Hospital of Nanjing Medical University, Nanjing, Jiangsu China; 2 Clinical and Translational Research Center for 3D Printing Technology, Shanghai Key Laboratory of Orthopaedic Implants, Department of Orthopaedic Surgery, Shanghai Ninth People’s Hospital, Shanghai Jiao Tong University School of Medicine, Shanghai, China; 3 Department of Nephrology, Affiliated Hospital of Nanjing Medical University, North District of Suzhou Municipal Hospital, Suzhou, China

**Keywords:** Regenerative medicine, Rheumatic diseases, Stem-cell biotechnology

**Dear Editor,**

Human cell-based and personalized in vitro cartilage models are urgently needed for osteoarthritis treatment in pre-clinical regenerative medicine development. Cellular self-assemblies and condensations of the appropriate stem cells could initiate the formation of transient tissue structures programmed for specific organogenesis processes.^1^ This recapitulation of developmental events has previously been demonstrated for the formation of cardiac, epithelial and liver organoids. However, there has been very limited progress in the development of human cartilage organoids for osteoarthritis (OA).^2^ Here, we describe the fabrication of functional bioengineered cartilage organoid suitable for OA treatment.

Briefly, agarose microwell inserts for formation of a high number of synovial mesenchymal stromal cell (SMSC) organoids with homogeneous size distribution were created as previously described by Leijten et al.^3^ 3D-cultured SMSC organoids were generated and phenotypically analyzed for potential applications in OA modeling and treatment (Fig. 1a). SMSCs self-assembled to form a stack of cells to attain a spheroid shape in the 4-week cultivation (Fig. 1b**;** Supplementary Fig. S1a), demonstrating compaction of the organoids with a confined actin cytoskeleton network (Fig. 1b). Chondrogenesis was defined for the organoids (Supplementary Fig. S1, b-e), showing significantly greater expression of chondrogenic markers SRY-related high mobility group-box gene 9 (SOX9) and aggrecan (ACAN) compared to the 2D control group (Supplementary Fig. S1, b–d). Meanwhile, the organoid lysates yielded a cartilaginous matrix that stained positively for toluidine blue, indicative of the formed proteoglycan-rich, cartilage-like extracellular matrix (ECM) (Supplementary Fig. S1e). Lacuna formation, a typical sign of cartilage formation, was observed for SMSC-organoids after transplantation subcutaneously in nude mice (Supplementary Fig. S1f), which generated substantial amounts of glycosaminoglycan (GAG) in cartilage tissues. Immunohistochemical analysis showed that the cartilage generated by SMSC-organoids deposited rich GAG (Supplementary Fig. S1, g-h) and expressed abundant ACAN and type II collagen (Fig. 1h), confirming better committed chondrogenic lineage (Supplementary Fig. S1, i-j). These results suggest that SMSC-organoids could generate ectopic cartilaginous tissue in vivo, indicating its potential in cartilage regeneration and OA therapy.

**Fig. 1:**
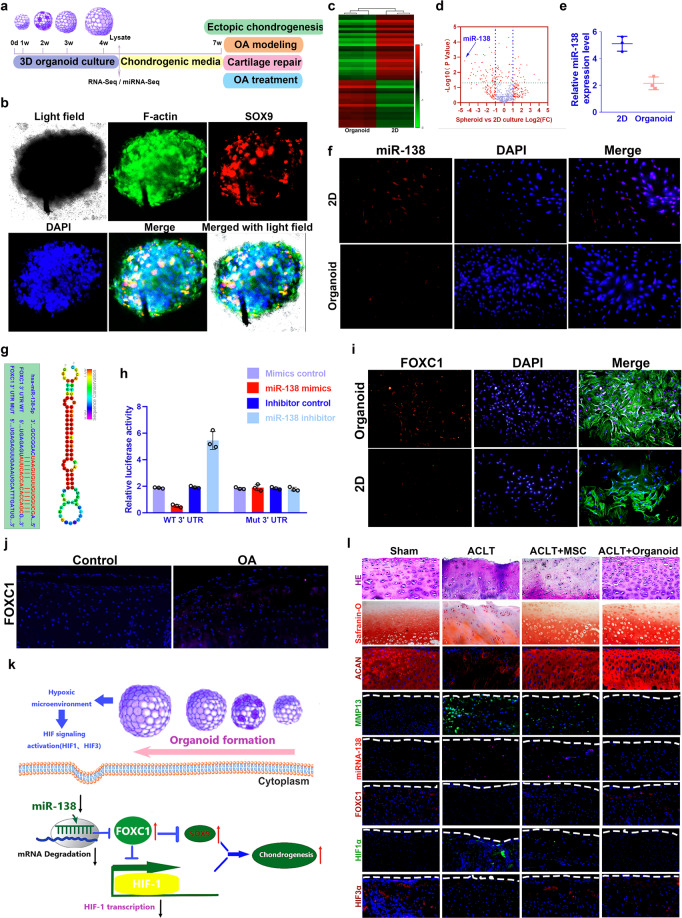
Generating 3D-cultured organoids for pre-clinical modeling and treatment of degenerative joint disease. **a** 3D-cultured SMSC organoids were generated and phenotypically analyzed with miRNA and mRNA sequencing for potential applications in chondrogenesis and further OA modeling and treatment. **b** SMSCs self‐assembled to attain a spheroid shape in the 4-week cultivation in the SMSC organoids. Filamentous‐actin (F‐actin, green) and chondrogenic marker SOX9 (red) staining demonstrated chondrogenesis in the confined actin cytoskeleton network of SMSC organoids after 4 weeks. **c** Heatmap of clustering dysregulated miRNA expression profiles with microarray in SMSC organoids compared to 2D cultured control. **d** Volcano plot of miRNA expression profiles and miR-138 was most significantly downregulated in SMSC organoids. **e**–**f** Downregulation of miR-138 was further validated with (**e**) qRT-PCR and (**f**) fluorescence in situ hybridization (FISH) in SMSC organoids. (miR-138: red; nucleus: blue). **g** Sequence of wild type (WT) and mutant (Mut) FOXC1 binding sites for miRNA-138 (left) and conservation level of miR-138 sequence among species (right). **h** Luciferase reporter assay analysis results to confirm direct interaction between miR-138 and FOXC1 binding sites. Relative luciferase reporter activity was assessed for co-transfected FOXC1 WT (or Mut) with miR-138 mimics or inhibitor in cultured primary human SMSC cells. miRNA-138 mimics control and inhibitor control served as negative controls. **i**–**l** FOXC1 expression(red) with immunostaining (**i**) in cultured SMSC organoids (green for cytoskeleton) and (**j**) OA cartilage tissues. Sections were counterstained with DAPI for nucleus (blue). **k** Schematic representation of how miR-138/FOXC1/HIF signaling pathway might mediate chondrogenesis and the therapeutic effects of SMSC organoids in OA. On the basis of described findings, hypoxia microenvironment of the SMSC organoids could downregulate miR-138 expression. miR-138 inhibition upregulated FOXC1 expression, and FOXC1 further suppressed HIF1α transcription and upregulated HIF3α expression, which attenuates the binding of HIF1α to its target genes and hence inhibits HIF1α transcription. **l** Histological assessments of joint cartilage with HE (1st row), safranin-O staining O (2nd row) and immunostaining for ACAN, MMP13, miR-138, FOXC1, HIF1α and HIF3α in different groups. Sham: no surgery group; ACLT: OA model group with anterior cruciate ligament transection; ACLT + MSC: only SMSC was injected for OA treatment; ACLT + organoid: SMSC organoids were injected for OA treatment

Main difference of the organoid we generated is its 3D shape and its firm stacking of cells. We presume that cellular communication would be altered in 3D organoid with modulated miRNA profiles in 3D organoid to mediate cellular communication. We sought to explore the miRNA expression profiles with miRNA microarray on three SMSC organoids vs. three 2D cultured SMSC samples. miR-138 expression was most significantly downregulated in SMSC organoids (Fig. 1c–e), which was further validated with qRT-PCR and fluorescence in situ hybridization (FISH) (Fig. 1e, f). To explore the role of miR-138 in OA development, miRNA microarray was performed on three OA cartilage samples from clinical OA patients vs. three control samples from patients with traumatic amputation (Supplementary Fig S2, a–b). miR-138 expression was significantly elevated in OA samples and further validated in another independent 12 OA samples vs. 6 controls. (Supplementary Fig. S2, c–d) Moreover, the expression of miR-138 in cartilage tissues from OA patients was correlated with the joint degeneration grade (*n* = 21; *r* = 0.73, *p* < 0.001; Supplementary Fig. S2e). No significant difference was observed between OA and controls with respect to miR-138 level in the synovial tissues (Supplementary Fig. S2f). These findings suggest that miR-138 might mediate the better chondrogenic properties of SMSC organoids and have cartilage-specific effects in OA development. Dysregulated mRNAs were also identified in SMSC organoids (Supplementary Fig. S3, a–c). Forkhead box C1 FOXC1 was identified as the target of miR-138 (Fig. 1g, Supplementary Fig. S3, d–e). To further confirm the functional interaction between miR-138 and FOXC1, we performed luciferase reporter assay analysis. Co-transfected FOXC1 Wild Type (WT) with miR-138 mimics in cultured primary human SMSC cells was significantly lower than relative luciferase reporter activity of cells transfected FOXC1-mut (mutant) with miR-138 mimics (Fig. 1g, h). This effect was further supported by gene expression in cultured SMSC organoids and OA cartilage tissues (Fig. 1i, j; Supplementary Fig. S3, f–g). These results validated FOXC1 as a direct target of miR-138.

To identify the altered downstream pathways mediated by miR-138/FOXC1 signaling in SMSC organoids, enriched Kyoto Encyclopedia of Genes and Genomes (KEGG) pathways in SMSC organoids were analyzed. Hypoxia induced factor (HIF) signaling pathway was significantly enriched for SMSC organoids in KEGG pathways (Supplementary Fig. S4a). Moreover, miR-138 inhibitor activated the expression of another subunit of hypoxia inducible factor-3α (HIF3α) HIF3α was reported to attenuate the transcription ability of HIF1α on its downstream target genes. Furthermore, FOXC1 small interfering RNA had effects on ACAN, SOX9, matrix metallopeptidase 13 (MMP13), vascular endothelial growth factor A, hypoxia inducible factor-1α (HIF1α) and hypoxia inducible factor-3αa (HIF3α) genes similar to the effects induced by miR-138 (Supplementary Fig. S4b), indicating that miR-138 regulates chondrogenesis and OA progression by targeting the FOXC1/HIF pathway. These results indicate that miR-138-mediated chondrogenesis in OA is primarily through the FOXC1/HIF pathway. miR-138 inhibition upregulated FOXC1 expression, and FOXC1 further suppressed HIF1α transcription and upregulated HIF3α expression (Supplementary Fig. S4, b–d). Meanwhile, HIF3α could attenuate the binding of HIF1α to the hypoxia-responsive elements (HRE) located within the enhancer/promoter of hypoxia-inducible target genes and hence inhibits HRE-driven transcriptional activation (Fig. 1k).

SMSC-organoids demonstrated chondrogenic properties and generated ectopic cartilaginous tissues in vivo. Meanwhile, downregulation of miR-138 was also previously reported as a protective factor in OA development. In this case, we hypothesize SMSC organoid might be a novel OA therapy with its modulation of the miR-138/FOXC1/HIF axis. To determine whether SMSC organoids transplantation would reduce or reverse the progression of OA, intra-articular injection of GFP-labeled SMSC-organoids was performed for rats with ACLT surgery (Supplementary Fig S5, a–b). Compared to control group with severe cartilage erosion, osteophyte formation and synovial inflammation, local delivery of SMSC or its organoids remarkably protected the structure of joint cartilage as determined by histological assessments and proteoglycan depositions (Fig. 1l; Supplementary Fig. S5c). No significant SBP difference was observed in subchondral bone (Supplementary Fig. S5c). Moreover, expression of MMP13 was significantly decreased by SMSC organoids transplantation, whereas an increase in ACAN expression was noted (Fig. 1l), indicating the restoration of balance in chondrocyte anabolism and catabolism by SMSC organoids. In evaluating the modulation of the miR-138/FOXC1/HIF signaling axis in OA and SMSC organoids transplantation, upregulation of miR-138 and HIF1α was observed with immunostaining in the cartilage tissue from control OA mice and inhibited in the following SMSC organoids treatment (Fig. 1l). Furthermore, in contrast to the decreased expression level of HIF3α and the miR-138 target gene FOXC1 in OA cartilage, SMSC organoids transplantation effectively elevated FOXC1 and HIF3α expression (Fig. 1l). Taken together, these results provided promising evidence that intra-articular SMSC organoids transplantation might be an effective therapeutic option for the prevention of OA, highlighting the miR-138/FOXC1/HIF signaling axis as a potential therapeutic target in the treatment of OA.

Our analysis led to the identification of miR-138/FOXC1/HIF signaling axis as a novel therapeutic target required for chondrogenesis and OA treatment by the SMSC organoids. In the present study, generation of SMSC organoids led to decreased miR-138 expression and higher expression of its target gene FOXC1, leading to better chondrogenic and anti-arthritic properties of the SMSC organoids compared to 2D cultured SMSCs. FOXC1 is highly expressed in non-OA cartilage samples. Previously, it has been shown that FOXC1 is required for human skeletal growth and chondrocyte maturation.^4^ Our results demonstrated that upregulation of FOXC1 in the transplanted SMSC organoids in OA model could regulate the HIF signaling pathway in the joint cartilage by activating HIF3α expression and inhibiting HIF1α expression. Overexpression of HIF1α has been well acknowledged in OA development, causing cartilage ECM catabolism and chondrocyte hypertrophy.^5^

In conclusion, the described SMSC organoids provide an alternate engineering approach for OA modeling and treatment. Creation of OA patient-specific SMSC organoids could design an in vitro organotypic disease model of OA progression with patient-specific genetic pathological stimuli. All together our data will pave the road to use 3D organoids-based models for tailored modeling and treatment of joint diseases.

## Supplementary information

Supplementary Materials

Supplementary Figure 1

Supplementary Figure 2

Supplementary Figure 3

Supplementary Figure 4

Supplementary Figure 5

## Data Availability

All data needed to evaluate the conclusions in the paper are present in the paper and/or the Supplementary Materials.
