# Supplementary material for: Generating 3D-cultured organoids for pre-clinical modeling and treatment of degenerative joint disease
Source: Signal Transduct Target Ther. 2021 Nov 12;6:380. doi: 10.1038/s41392-021-00675-4 (PMC8585871; doi:10.1038/s41392-021-00675-4)
Supplement: Supplementary file 1 — Supplementary Materials [file 41392_2021_675_MOESM1_ESM.docx]

Supplementary Materials for

**Generating 3D-cultured organoids for pre-clinical modeling and treatment of degenerative joint disease**

Ye Sun^1,2,*^, Qiang Wu^2^, Kerong Dai^2^, Yongqing You^3^, Wenbo Jiang^2^

Correspondence to: sunye881005@163.com

**This PDF file includes:**

Materials and Methods

Figures. S1 to S5

**Materials and Methods**

**Patient samples:** Joint samples of cartilage and synovial tissues were obtained from 6 control patients(39.6 ± 7.1 years) with leg amputation and 21 OA patients (42.3 ± 4.8 years) undergoing arthroscopy or arthroplasty. OA severity was calculated with OARSI scores for OA patients as described. The study protocol was approved by the Ethics Committee of shanghai ninth Hospital, and signed informed consent were acquired from enrolled patients and controls.

**Cell Expansion:** Human SMSCs and chondrocytes were isolated from synovial biopsies of healthy clinical donors. The SMSC pools were expanded until passage 3 for organoid formation at 37 °C, 5% CO2, and 95% humidity in Dulbecco’s modified Eagle medium (DMEM, Life Technologies, China) with 10% fetal bovine serum (HyClone FBS, Thermo Scientific, USA), 1% antibiotic– antimycotic (100 units mL^−1^ penicillin, 100 mg mL^−1^ streptomycin, and 0.25 mg mL^−1^ amphotericin B), and 1 × 10^−3^ M sodium pyruvate (Life Technologies, China). Medium was changed every 2–3 days, and SMSCs were harvested with Trypsin (Life Technologies, China) at a confluence of 80–90%. Trypsin was used for all passaging and harvesting steps during cell handling. The ethical committee for shanghai ninth hospital (school of medicine, Shanghai jiaotong university) approved all procedures, and patients’ informed consent forms were obtained.

**Formation of SMSC organoids:** Agarose microwell inserts for formation of a high number of SMSC organoids with homogeneous size distribution were created as previously described by Leijten et al.^1^ Briefly, 3 % (w/v) Agarose (Invitrogen, China) was poured onto a polydimethylsiloxaan (PDMS, Corning Sylgard 184 elastomer, MAVOM Chemical Solutions) master mould containing 200 µm-diameter pillars. The agarose was let to solidify where after microwell inserts with an area of ≈1.8 cm^2^ were punched out, placed in 24-well plates, 1 mL of phosphate-buffered saline (PBS) was added and the wells were sterilized under UV for 30 min. Each well insert contained near 2000 microwells. SMSCs were harvested and seeded with a concentration of 1000 000 cells per well to obtain ≈500 cells per organoid after self-aggregation. SMSC organoids were differentiated into cartilaginous microtissues in a serum-free chemically defined chondrogenic medium (CM) containing LG-DMEM (Gibco) supplemented with 1% antibiotic–antimycotic (100 units mL^−1^ penicillin, 100 mg mL^−1^ streptomycin, and 0.25 mg mL^−1^ amphotericin B), 1 × 10^−3^ M ascorbate-2 phosphate, 100 × 10^−9^ M dexamethasone, 40 µg mL^−1^ proline , ITS+ Premix Universal Culture Supplement (Corning) (including 6.25 µg mL^−1^ insulin, 6.25 µg mL^−1^ transferrin, 6.25 µg mL^−1^ selenious acid, 1.25 µg mL^−1^ bovine serum albumin (BSA), and 5.35 µg mL^−1^ linoleic acid), 100 ng mL^−1^ growth/ differentiation factor 5 (GDF5) (PeproTech) and 10 ng mL^−1^ TGF-β3 (PeproTech),^2^ Half of the media volume was changed every 2-3 days. Cell viability in SMSC organoids was assessed qualitatively with LIVE/DEAD Viability/Cytotoxicity Kit (Invitrogen) by following the manufacturer’s protocol as previously reported. Cell nucleus and F-actin distribution within SMSC organoids was visualized by staining with 2.5 µg mL−1 4′,6-diamidino-2-phenylindole (DAPI) (Invitrogen) and 0.8 U mL^−1^ Alexa Fluor 488 phalloidin (Invitrogen) during 1 h at room temperature. Stained organoids were imaged with an inverted laser scanning fluorescence confocal microscope with 1 µm thick slices as previously reported.^1^

**Microarray analysis:** Microarray analysis of 6 cell samples (miRNA and mRNA microarray for 3 SMSC organoids and 3 2D-culture SMSCs) and 6 cartilage samples (miRNA microarray for 3 OA and 3 controls) was performed using an Agilent mRNA and miRNA Microarray Kit, Release 21.0, 8x60K (Agilent Technologies, CA, USA). Total RNA was quantified by a NanoDrop ND-2000 (Thermo Scientific, SA, USA), and RNA integrity was assessed using an Agilent Bioanalyzer 2100 (Agilent Technologies). Sample labeling, microarray hybridization and washing were performed according to the manufacturer’s protocols. Briefly, total RNA was dephosphorylated, denatured and then labeled with cyanine-3-CTP. After purification, the labeled RNAs were hybridized to the microarray. After being washed, the arrays were scanned with an Agilent Scanner G2505C (Agilent Technologies). Feature Extraction software (version 10.7.1.1, Agilent Technologies) was used to analyze the array images to obtain raw data. Next, Genespring software (version 14.8, Agilent Technologies) was utilized to finish the basic analysis of the raw data. First, the raw data were normalized with the quantile algorithm. The probes that had at least 100.0 percent of samples in any 1 condition out of 2 conditions with flags in "Detected" were chosen for further data analysis. Differentially expressed miRNAs and mRNAs were then identified using R software (Version 3.6.1) with the “limma” package through fold change (FC) and adjusted P value. The threshold for up- and down-regulated genes was set at an FC > 4.0 and an adjusted P value < 0.05. The differentially expressed miRNAs were further validated by quantitative reverse transcriptase–polymerase chain reaction (qRT-PCR). Target genes of the candidate miRNA were identified based on the intersection between differentially expressed mRNAs and different predicted algorithms (Funrich software and 5 online predicting databases including miRDB, miRanda, TargetScan, PITA, and PicTar). Gene ontology (GO) analysis and Kyoto Encyclopedia of Genes and Genomes (KEGG) analysis were applied to determine the roles of these target genes by R. Hierarchical clustering was performed to show the differential miRNA and mRNA expression patterns among samples using scatter plots, volcano plots and heatmaps for visualization.

**Animal experiments**

**Ectopic cartilage formation in vivo:** The animal experiment protocols were approved by Shanghai Ninth Hospital, medical school of Shanghai Jiao Tong University Ethics Committee and the local Institutional Animal Care and Use Committee (IACUC) and complied with the Guide for the Care and Use of Laboratory Animals published by the National Academy Press (National Institutes of Health Publication No. 85-23, revised 1996). SMSCs or induced SMSC organoids were suspended in α-MEM containing 10% FBS. Then, 100 μl of the cell suspension was injected subcutaneously into the dorsal flank of 6-week old female nude mice. Mice were sacrificed after 4 weeks, and the injected sites were dissected from the mice. The samples were fixed in 4% paraformaldehyde, processed, and embedded in paraffin. Serial sections (4-μm thick) of the generated ectopic cartilage were cut through the center of the injection site and stained with alcian blue toluidine blue and safranin-O according to standard protocols. Immunohistochemical staining of chondrocyte markers ACAN and COL2A1 was conducted according to standard protocols in the generated cartilage tissue sections in different groups. The stained images were taken using a light microscope. GAGs and types II and X collagen were quantitatively assayed (6 vs 6) and normalized to DNA content. GAG production and COL II and X expression was compared among different treatment groups.

**Injection of SMSC mini-organoids for OA treatment in rat knee joint:** Rats were used to examine the effect of SMSC mini-organoids in a OA model in vivo. After the skin incision, a 3-cm medial parapatellar incision was applied and the patella was dislocated. Anterior cruciate ligament transection was performed to construct an OA model. Rats were randomized into four groups (n=6 for each group; two knees of each rat were used): ACLT group with no treatment, ACLT+MSC group with 2d-cultured SMSC injection, ACLT + organoid group with mini-organoid injection and the sham group with sham surgery. After the operation, rats were allowed to move freely in their single cages and fed with standard food and water. Synovial fluid of the operated knee joint was collected at different time points. Serial sections (4-μm thick) were cut sagittally through the center of the most diseased osteoarthritic site and stained with H&E and Safranin-O & fast green according to standard protocols. Immunohistochemical staining of cartilage markers (ACAN and MMP13) and markers in the miR-138/FOXC1/ HIF signaling axis(miRNA-138, FOXC1, HIF1α and HIF3α) were also conducted according to standard protocols in the generated cartilage tissue sections in different groups compared to the native cartilage. The stained images were taken using a light microscope. Histological assessment of sagittal sections of the knee joints was conducted by two blinded observers who followed the Osteoarthritis Research Society International (OARSI) scoring system.^3^ Measurements were also performed for osteophyte maturation, synovitis score (0-3, 0 = no synovial thickening; 1 = lining of two cell layers; 2 = several extra cell layers; 3= clear inflammation with cell infiltrate or exudate) and subchondral bone plate thickness (region between the osteochondral junction and marrow space on the medial side of the tibial plateau) using Bioquant Osteo software (BIOQUANT, Inc.) as describe previously.^4,5^

**Immunofluorescence staining of histological sections:** To evaluate the distribution and expression of proteins, immunofluorescence analysis was performed as described by previous reports ^6^. In brief, frozen sections of mice colons with a thickness of 6μm were fixed in 1% paraformaldehyde and washed using PBS. After blocking with 5% normal goat serum diluted in PBS, sections were then incubated with primary antibodies in PBS with 1% goat serum (4℃, overnight). After washing with PBS, sections were incubated with secondary antibodies for 1 h. Images visualization was performed using a confocal microscopy (Olympus, Tokyo, Japan). The primary antibodies in this study were available upon reasonable request.

**Western Blotting:** As described by previous studies, western blotting analysis was performed to assess expressions of proteins ^7^. Briefly, protein lysates were prepared from SMSCs or ATDC5 using RIPA lysis buffer containing phosphatase inhibitors and protease. The protein concentrations determination was performed using a BCA Protein Assay Reagent Kit (Pierce Biotechnology, Rockford, IL, USA). 15 μg of total protein was resolved by sodium dodecyl sulfate polyacrylamide gel electrophoresis (SDS-PAGE), and then transferred onto the polyvinylidene fluoride (PVDF) membrane. After being blocked by 5% non-fat dry milk in Tris-buffered saline for 1 h (room temperature), the membranes were incubated with primary antibodies. After incubation with horseradish peroxidase (HRP)-conjugated species-matched secondary antibodies, the blots were then visualized using chemiluminescence kits (Amersham Corp, Buckinghamshire, UK). The densitometric analysis of blots was performed using ImageJ software (Media Cybernetics, Silver Spring, MD, USA). GAPDH (Abcam) was utilized as the normal control.

**RNA isolation, cDNA synthesis, and qRT-PCR:** Total RNA isolation from joint tissues or SMSC cells was performed using TRIzol reagent (Invitrogen, Carlsbad, CA, USA) according to the manufacturer’s instructions. RNA quality and quantity determination were performed using a bioanalyzer (Agilent Inc., Santa Clara, CA, USA) and nanodrop (Thermo Scientific). For quantitative detection of miRNA and mRNA, RT-PCR was performed using a qSYBR-green-containing PCR kit (Qiagen, Germantown, MD, USA) with an RT-PCR system (Applied Biosystems, SA, USA). U6 small nuclear RNA (snRNA) and GAPDH were used as controls for normalization. mRNA and miRNA qRT-PCR primers and internal control were purchased from Applied Biosystems. All PCR assays were performed in triplicate using 2^-△△Ct^ method. All Primer sequences are available from the authors upon request.

**In vitro miRNA and siRNA transfection:** Human SMSCs and chondrocytes were transfected with miRNA-138 mimics, inhibitor, or negative control using Lipofectamine RNAiMAX transfection reagent (Invitrogen). Cells were transfected with FOXC1 siRNA, HIF3α siRNA or control siRNA (Thermo Scientfic) using Lipofectamine 3000 (Invitrogen) according to the manufacturer's instructions. The FOXC1 expression plasmid was obtained using the pcDNA™3.1/V5-His TOPO™ TA Expression Kit (Invitrogen™). After 48 h of transfection, the expression levels of the target genes were evaluated from the collected cellular lysates by qRT-PCR and western blotting.

**3′-Untranslated region (UTR) cloning and luciferase assay:** To construct the FOXC1 3′-UTR-Luc reporter plasmid, a wild-type (Wt) or mutant (Mut) fragment of the 3′-UTR of FOXC1 containing the predicted miR-138 binding sites was PCR-ampliﬁed and inserted into the psi-CHECKTM-2 vector (Promega, Madison, WI). To conduct the luciferase assay, human SMSCs and chondrocytes were seeded into a 96-well plate and co-transfected with WT- or Mut-FOXC1 3′-UTR-Luc reporter plasmids and a miR-18 mimic, inhibitor or negative control with Lipofectamine PLUSTM reagent (Invitrogen, China). At 48 h after transfection, the cell lysates were harvested for luciferase activity determination using the Dual-Glo Luciferase Assay system (Promega, Madison, WI, USA) and were normalized to firefly luciferase activity. Each experiment was performed in triplicate and independently replicated three times.

**Cell immunofluorescence:** Cell immunofluorescence analysis was performed according to the descriptions by previous studies ^8^. Briefly, SMSCs or induced organoids were fixed in 4% PFA, permeabilized with PBS containing 0.5% Triton X-100 for 20 min, and then blocked with 3% BSA containing 0.025% Triton X-100 and 5% FBS at room temperature for 30 min. Cells were immunostained via incubation with primary antibodies at 4°C overnight. After that, the cells were washed using PBS three times and incubated with appropriate secondary antibodies. After the cells were washed, DAPI was used for nuclear counterstaining for 5 min. Immunfluorescence visualization was performed using a confocal microscope (Carl Zeiss, Germany). Each experiment was conducted in triplicate, and representative confocal microscopy images are shown.

**Fluorescence in situ hybridization (FISH):** FISH experiments were performed for cell samples from SMSC and SMSC organoids, and cartilage tissues from OA patients and controls. The frozen sample tissues were fixed in 4% PFA for 10 min and washed with PBS 3 times (5 min each time). After being digested in 20 µg/ml protease K for 15 min, the slides were prehybridized for 1 h at 37 °C and then incubated in 8 ng/µl Cy3-conjugated has-miR-138 probe (synthesized by Genscript, China) directed against the full length mature miR-138 sequence in the hybridization mixture at 37 °C overnight. Then, the slides were washed using 2× SSC (10 min) at 37 °C, 1× SSC (2×5 min) at 37 °C, and 0.5× SSC (10 min) at RT. The sections were then counterstained with DAPI and incubated for 8 min. The slides were observed with a NIKON biological microscope (Nikon Eclipse ci, Nikon Corporation, Tokyo, Japan) and imaged using an imaging system (Nikon DS-U3, Nikon Corporation, Tokyo, Japan). All the experimental procedures were conducted in triplicate within an RNase-free environment after DEPC processing.

**Statistical analysis:** The software of SPSS (Version 19.0, SPSS Inc., Chicago, IL, USA) and GraphPad Prism (Version 8.0, GraphPad Software Inc., San Diego, CA, USA) were used for statistical analysis. Mann–Whitney U test, Student’s t test and one-way anova test were used for data analysis as appropriate. Pearson’s correlation analysis was performed for the correlation between relative miR-138 expression and OA progression in clinical osteoarthritic patients. *P* < 0.05 was considered statistically significant.

**Reference**

1 Leijten, J. *et al.* Bioinspired seeding of biomaterials using three dimensional microtissues induces chondrogenic stem cell differentiation and cartilage formation under growth factor free conditions. *Sci Rep*. **6**, 36011, (2016).

2 Mendes, L. F. *et al.* Combinatorial Analysis of Growth Factors Reveals the Contribution of Bone Morphogenetic Proteins to Chondrogenic Differentiation of Human Periosteal Cells. *Tissue Eng Part C Methods*. **22**, 473-486, (2016).

3 Gerwin, N., Bendele, A. M., Glasson, S. & Carlson, C. S. The OARSI histopathology initiative - recommendations for histological assessments of osteoarthritis in the rat. *Osteoarthritis Cartilage*. **18 Suppl 3**, S24-34, (2010).

4 Zhen, G. *et al.* Inhibition of TGF-beta signaling in mesenchymal stem cells of subchondral bone attenuates osteoarthritis. *Nat Med*. **19**, 704-712, (2013).

5 Kim, J. H. *et al.* Regulation of the catabolic cascade in osteoarthritis by the zinc-ZIP8-MTF1 axis. *Cell*. **156**, 730-743, (2014).

6 Clayburgh, D. R. *et al.* Epithelial myosin light chain kinase-dependent barrier dysfunction mediates T cell activation-induced diarrhea in vivo. *J Clin Invest*. **115**, 2702-2715, (2005).

7 Ji, M. L. *et al.* Preclinical development of a microRNA-based therapy for intervertebral disc degeneration. *Nat Commun*. **9**, 5051, (2018).

8 Nguyen, H. T. *et al.* Crohn's disease-associated adherent invasive Escherichia coli modulate levels of microRNAs in intestinal epithelial cells to reduce autophagy. *Gastroenterology*. **146**, 508-519, (2014).


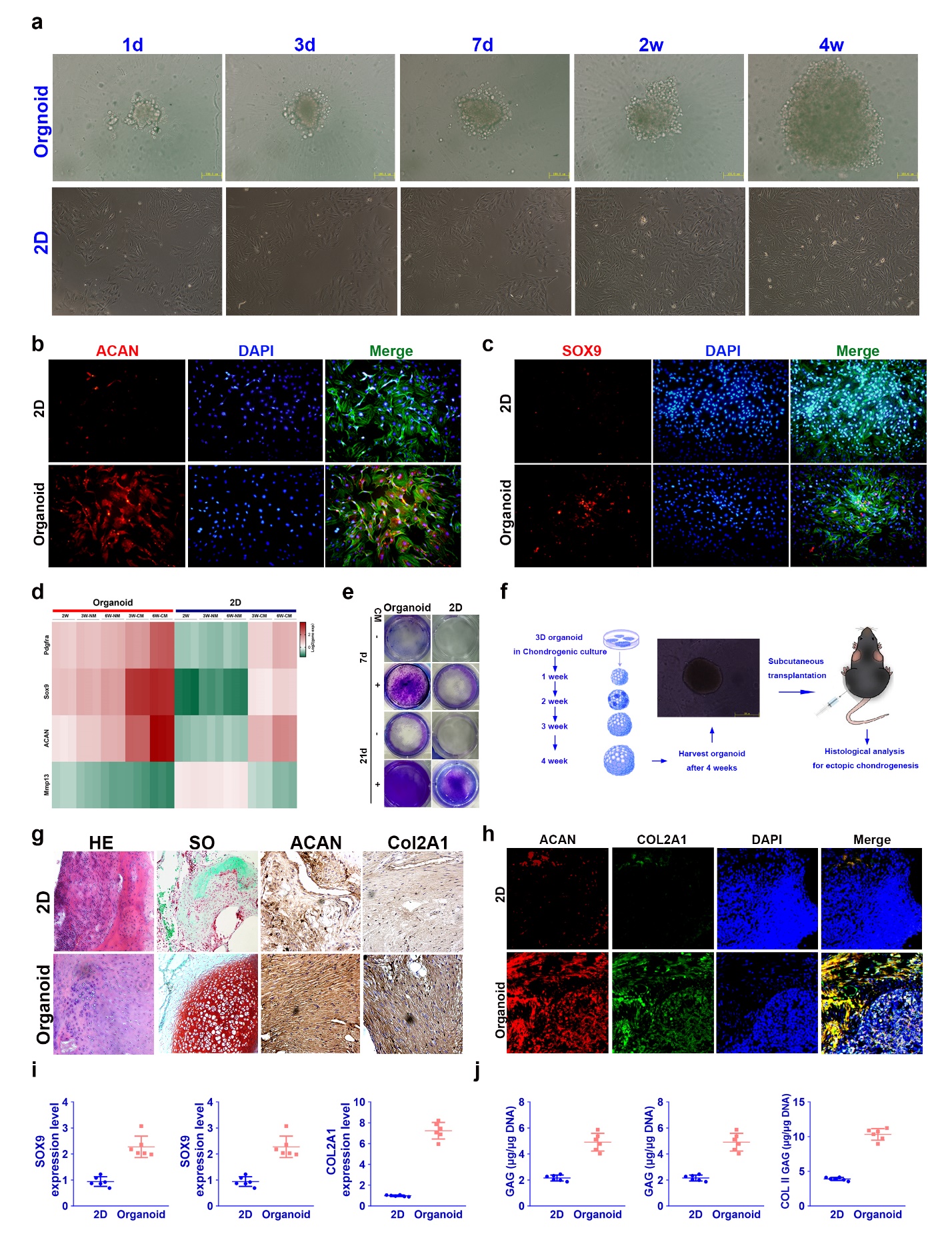


**Figure. S1. Generation of 3D-cultured SMSC organoids prompting chondrogenesis. a.** SMSCs self‐assembled to attain a spheroid shape in the four-week cultivation in the SMSC organoids. Filamentous‐actin (F‐actin, green) and chondrogenic marker SOX9 (red) staining demonstrated chondrogenesis in the confined actin cytoskeleton network of SMSC organoids after 4 weeks. **b-c.** Chondrogenesis was defined for organoids compared to 2D culture with immunostaining of ACAN and SOX9 (red) in vitro. Counter-staining with F-actin (green) and DAPI (blue) was applied. **d.** SMSC-organoids lysates were assayed with qRT-PCR for expression of chondrogenic markers PDGFRα, SOX9, ACAN and MMP13 compared to the 2D culture group. **e.** Chondrogenesis of SMSC organoid lysates yielded a cartilaginous matrix with toluidine blue staining. **f.** SMSC organoids were transplanted subcutaneously in nude mice for 4 weeks **g.** Histological examination of harvested SMSC-organoid derived cartilaginous tissue with HE and Safranin-O (SO) staining. Immunohistochemical analysis was also conducted with immunostaining of Chondrogenic markers ACAN and Col2A1. **h.** Immunofluorescent staining was also conducted with ACAN(red) and Col2A1(green) for the harvested cartilage tissues after subcutaneous implantation. Nucleus was stained with DAPI (blue). **i.** Cartilaginous tissues generated in vivo by SMSC organoids and 2D culture SMSCs were next analyzed with qRT-PCR for chondrogenic gene expressions. J. Quantification of deposited GAGs and collagens was also performed, confirming the better chondrogenic lineage committed by the SMSC-organoids.


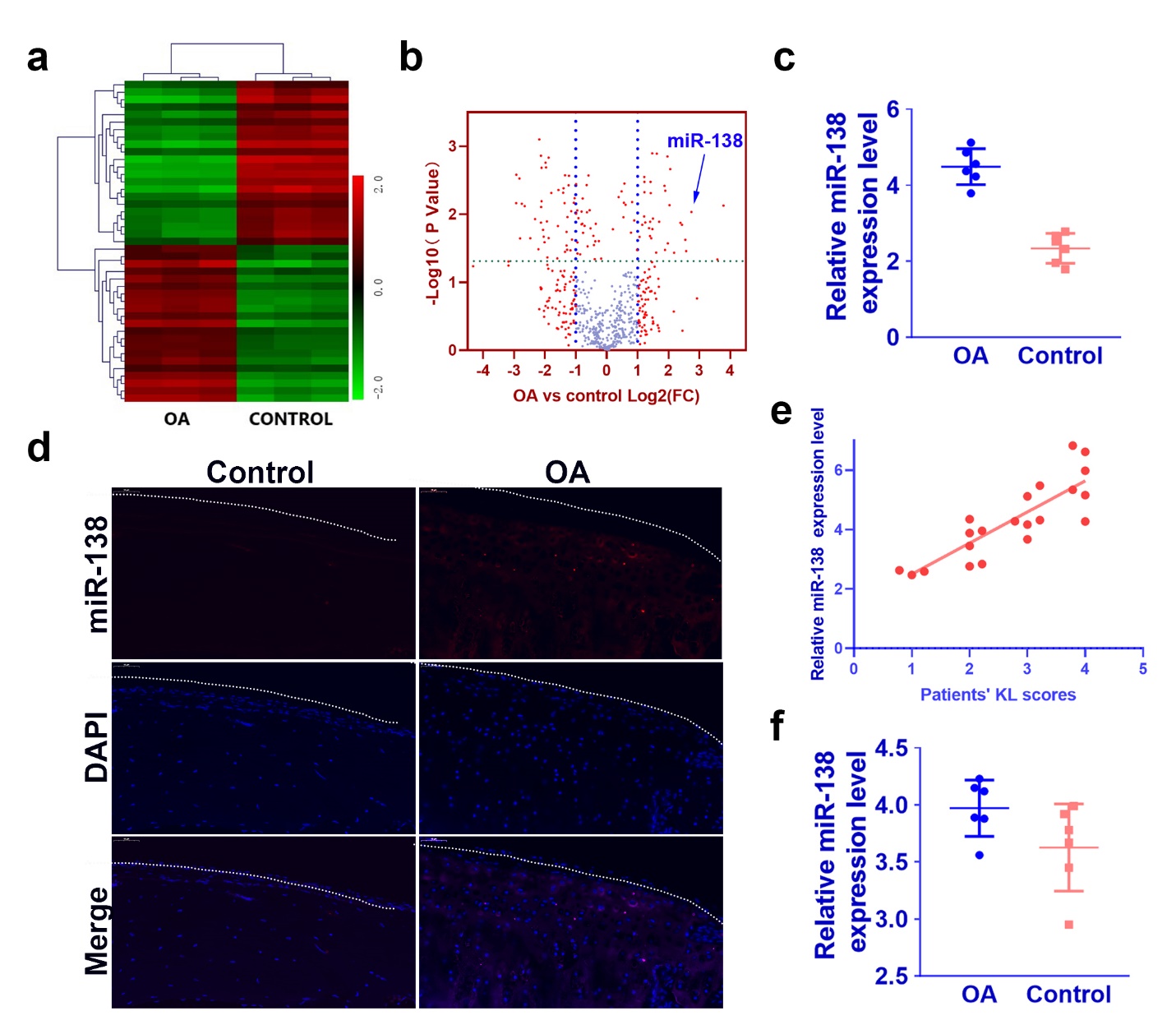


**Figure. S2. Discovery of organoid-specific miRNAs with microarray analysis. a.** Heatmap of clustering dysregulated miRNA expression profiles with microarray in three OA cartilage samples from clinical OA patients vs. three control samples. **b.** Volcano plot of miRNA expression profiles and miR-138 was significantly upregulated in OA samples. **c-d.** Upregulation of miR-138 was further validated with **c)** qRT-PCR and **d)** fluorescence in situ hybridization (FISH) in OA samples. (miR-138: red; nucleus: blue). **e.** Correlation between miR-138 expression levels and joint degeneration grade in cartilage tissues from OA patients (n=21; r=0.73, p < 0.001). **f.** Comparison of miR-138 expression level in the synovial tissues from OA patients and controls.


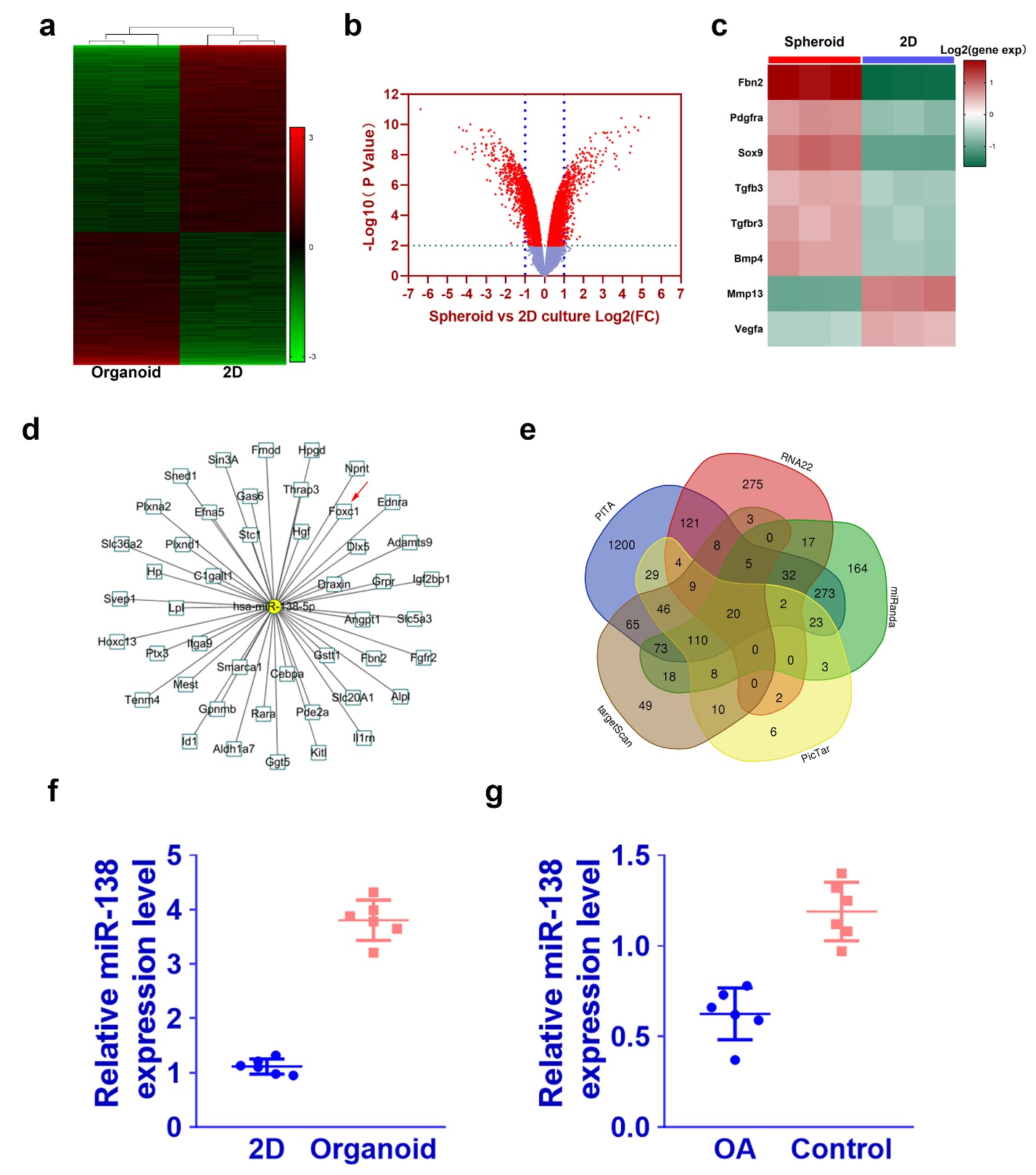


**Figure. S3. Identification of FOXC1 as a target gene of miR-138. a.** Heatmap of clustering dysregulated mRNA expression profiles with microarray in SMSC organoids compared to 2D cultured control. **b.** Volcano plot of mRNA expression profiles in SMSC organoids. **c.** Dysregulated typical chondrogenic markers derived from the microarray results for the SMSC organoids. **d.** miRNA–mRNA network using the Cytoscape software was constructed for the SMSC organoids. **e.** All predicted genes were compiled for Venn analysis to search the potential targets of miR-38. **f-g.** FOXC1 expression with qRT-PCR in cultured SMSC organoids and OA cartilage tissues.


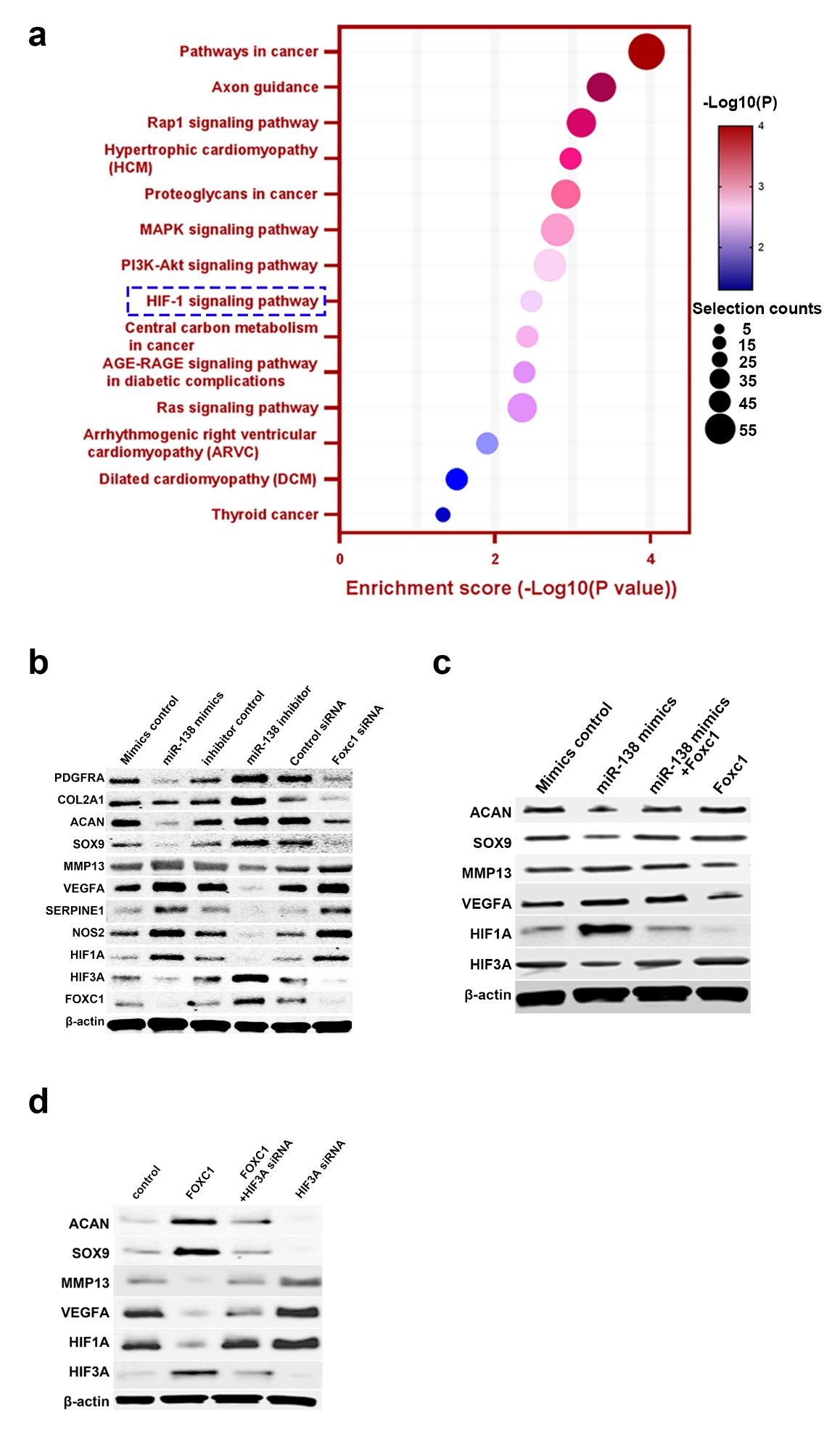


**Figure. S4. MiR-138 regulates chondrogenesis and OA development by modulating the FOXC1/HIF signaling pathway. a.** Significantly enriched pathways for SMSC organoids in Kyoto Encyclopedia of Genes and Genomes (KEGG) pathways. HIF signaling pathway was marked blue. **b.** Cultured primary human chondrocytes were transfected with miR-138 mimics, miR-138 inhibitor, their negative controls, control siRNA or FOXC1 siRNA for 72h respectively and the expression levels of chondrogenic markers PDGFRA, COL2A1, ACAN, SOX9, MMP13 and FOXC1/HIF signaling pathway markers VEGFA, SERPINE1, HIF1α, HIF3α, FOXC1 were assessed with western blot. β-actin served as control. **c.** Rescue experiments was established in cultured primary human chondrocyte to validate the relationship between miR-138 and FOXC1. Inhibition of ACAN and SOX9 expression levels by miR-138 mimics was rescued by restoration of FOXC1 expression. In comparison, inhibition of MMP13 and HIF1α expression levels by FOXC1 overexpression was rescued by miR-138 mimics. **d.** Upregulation of ACAN and SOX9 expression levels by FOXC1 was abolished by silencing of HIF3α expression. In comparison, upregulation of HIF1α target genes (MMP13 and VEGFA) expression levels by silencing of HIF3α was abolished by FOXC1 overexpression.


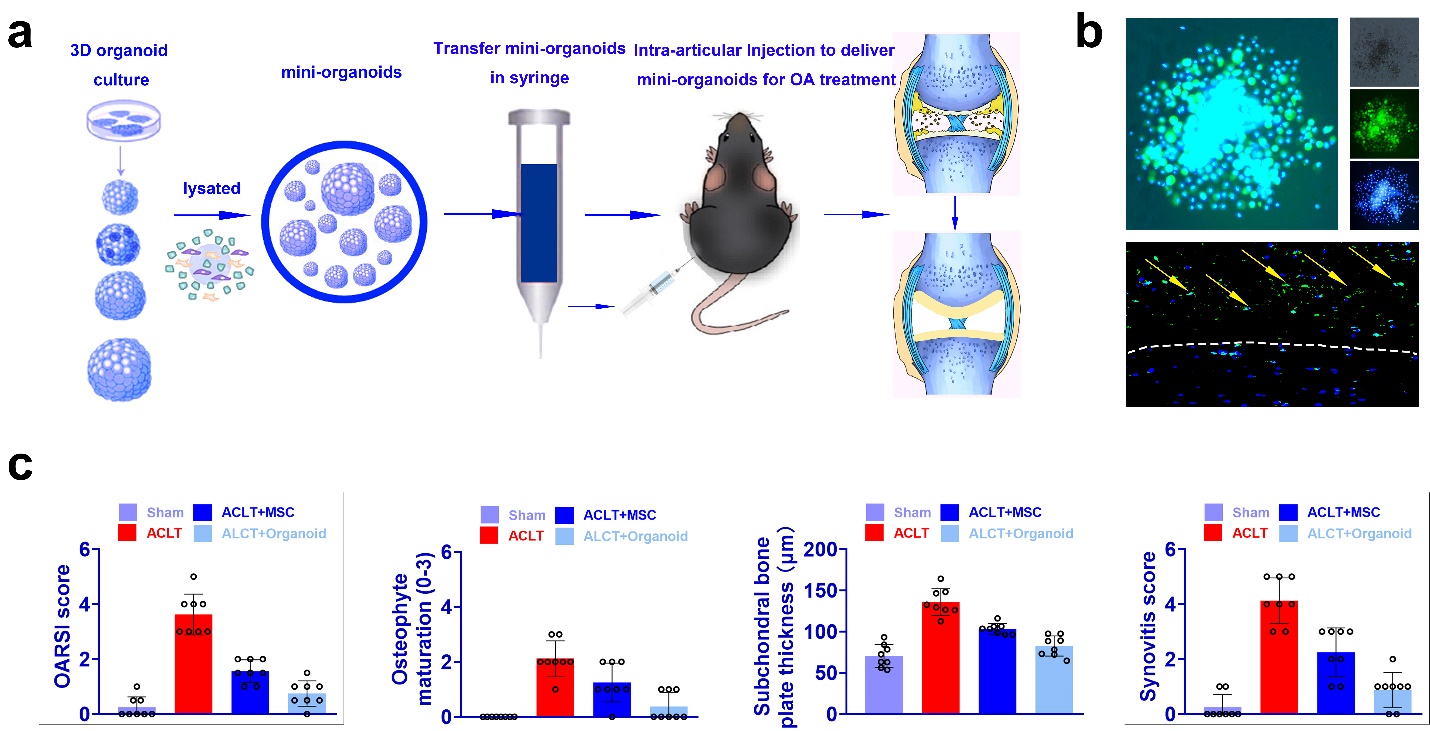


**Figure. S5. Intra-articular SMSC organoid transplantation reversed OA development by regulating the miR-138/FOXC1/HIF signaling axis. a-b.** To determine whether SMSC organoids transplantation would reduce or reverse the progression of OA, intra-articular injection of SMSC-organoids labeled with green fluorescence protein (GFP) was performed for rats with ACLT surgery. **b.** Delivery of the GFP-label SMSC organoids in vivo (yellow arrow) were monitored with GFP fluorescence (green) and showed an ideal intra-articular delivery effect. **c.** Quantification and comparison of histological grade for OA progression in different groups. Joint destruction severity was determined with OARSI score, osteophyte formation, subchondral bone plate thickness and synovial inflammation as previously reported.^3-5^ Sham: no surgery group; ACLT: OA model group with anterior cruciate ligament transection; ACLT+MSC: only SMSC was injected for OA treatment; ACLT+organoid: SMSC organoids were injected for OA treatment.
