# Supplementary figures and images for: Generating 3D-cultured organoids for pre-clinical modeling and treatment of degenerative joint disease
Source: Signal Transduct Target Ther. 2021 Nov 12;6:380. doi: 10.1038/s41392-021-00675-4 (PMC8585871; doi:10.1038/s41392-021-00675-4)

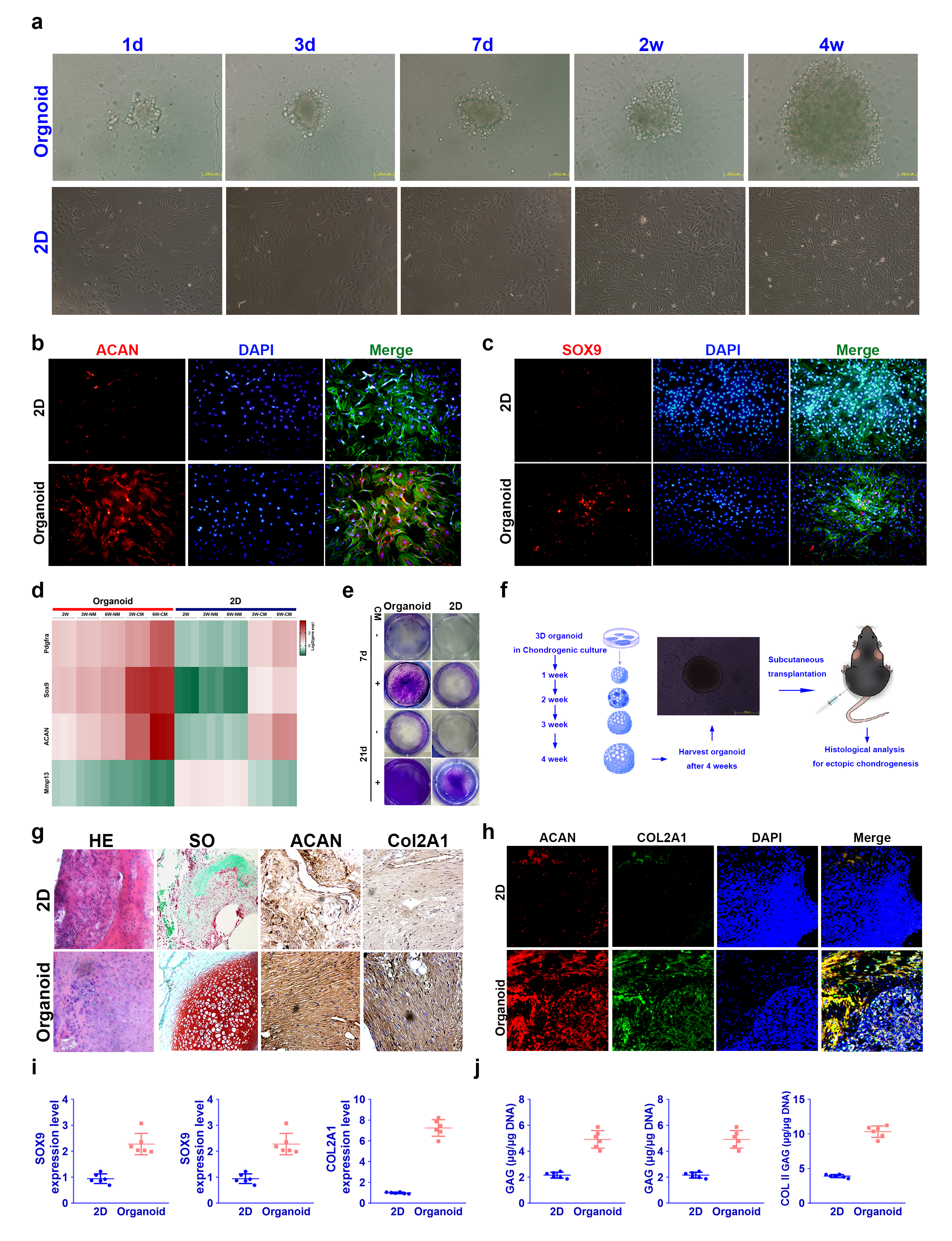

Supplement: Supplementary file 2 — Supplementary Figure 1 [file 41392_2021_675_MOESM2_ESM.jpg]

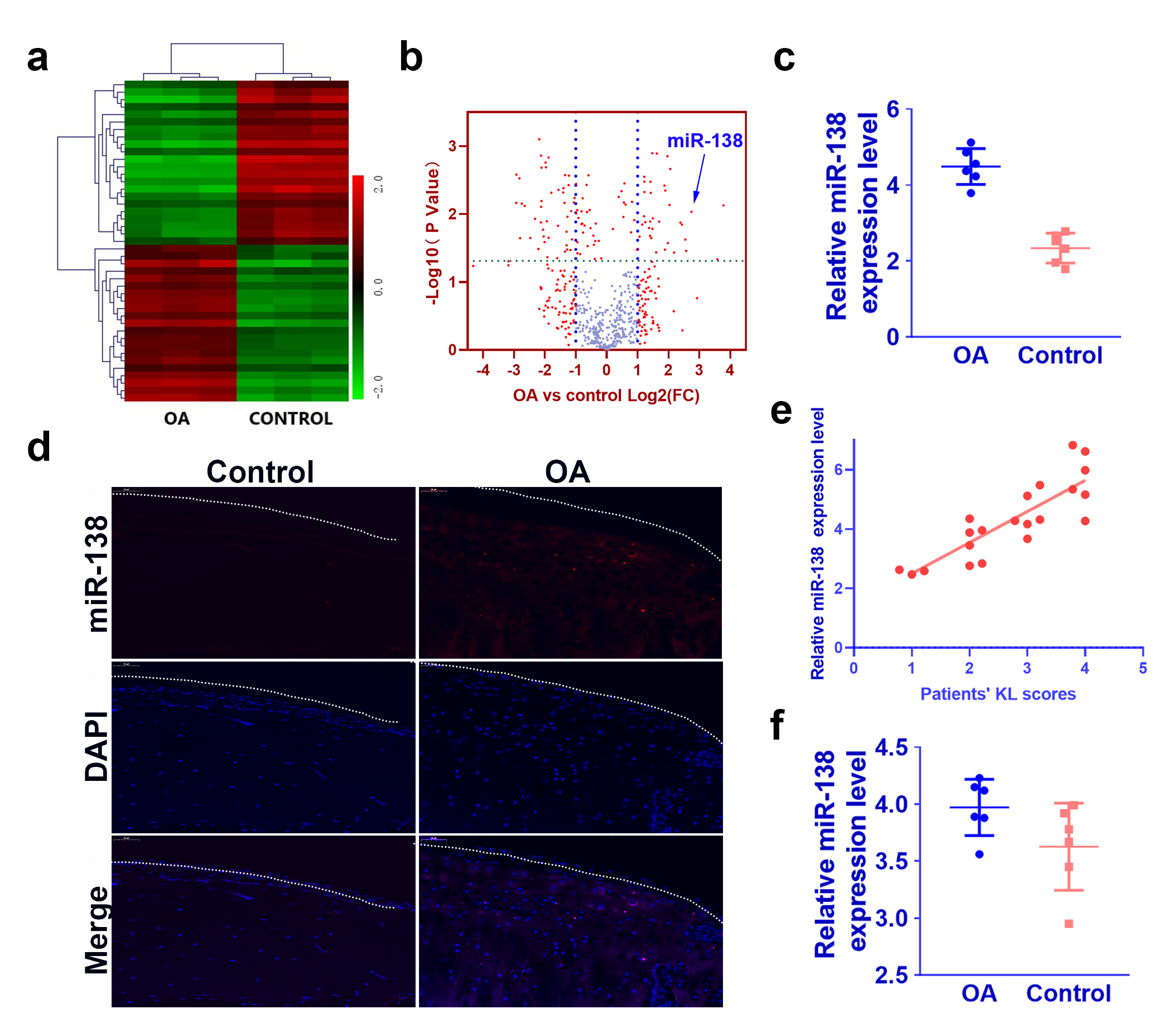

Supplement: Supplementary file 3 — Supplementary Figure 2 [file 41392_2021_675_MOESM3_ESM.jpg]

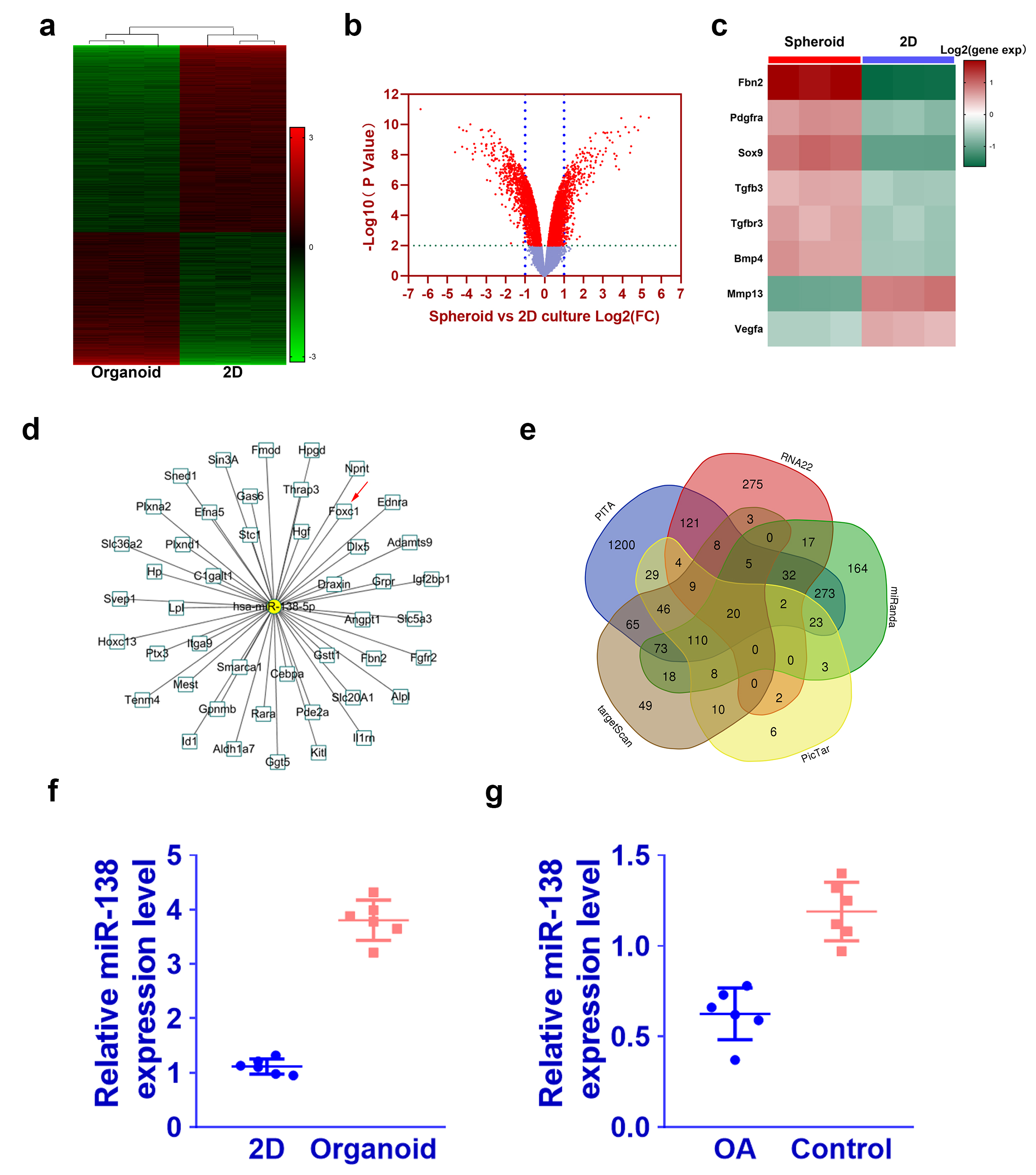

Supplement: Supplementary file 4 — Supplementary Figure 3 [file 41392_2021_675_MOESM4_ESM.jpg]

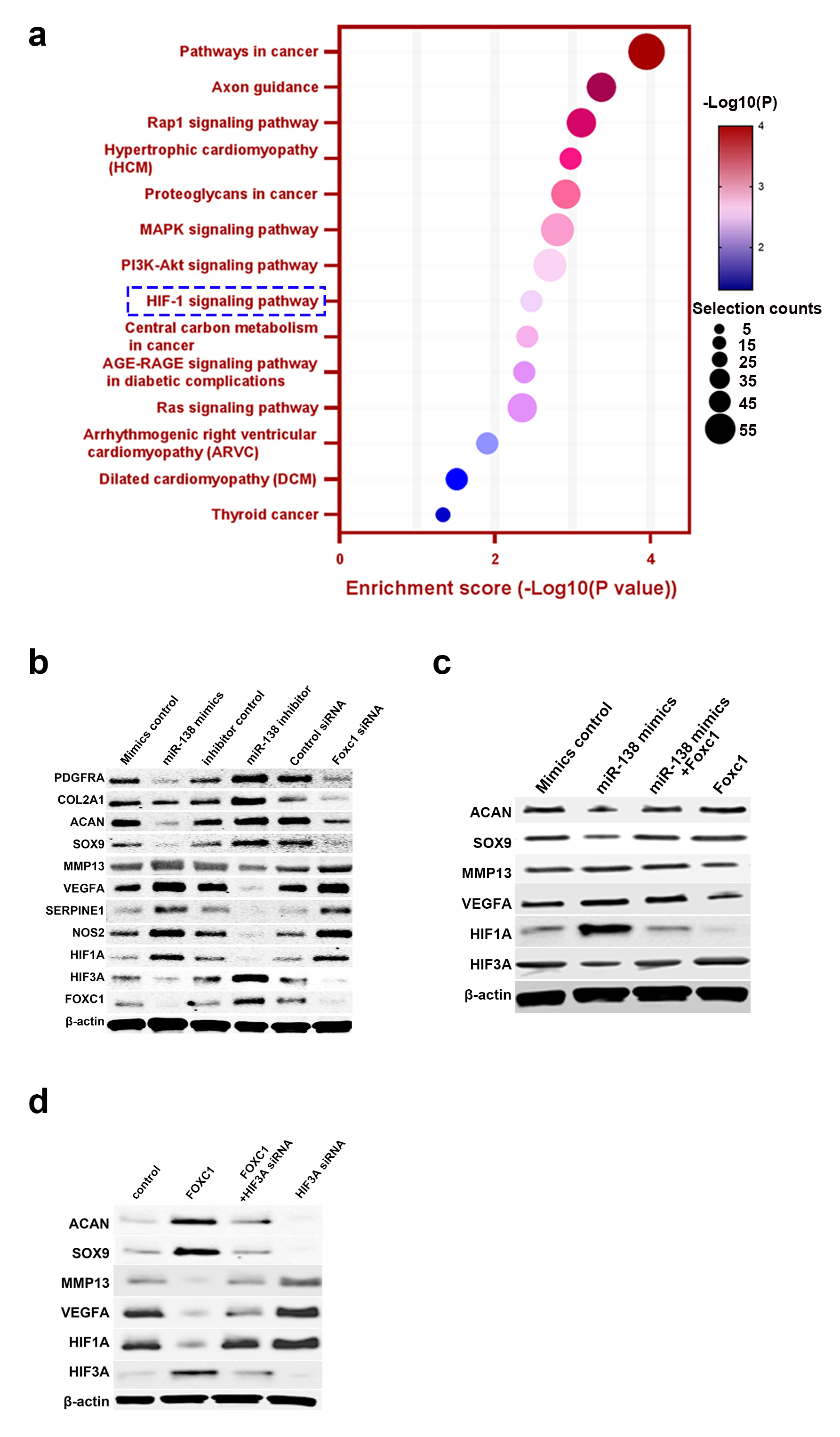

Supplement: Supplementary file 5 — Supplementary Figure 4 [file 41392_2021_675_MOESM5_ESM.jpg]

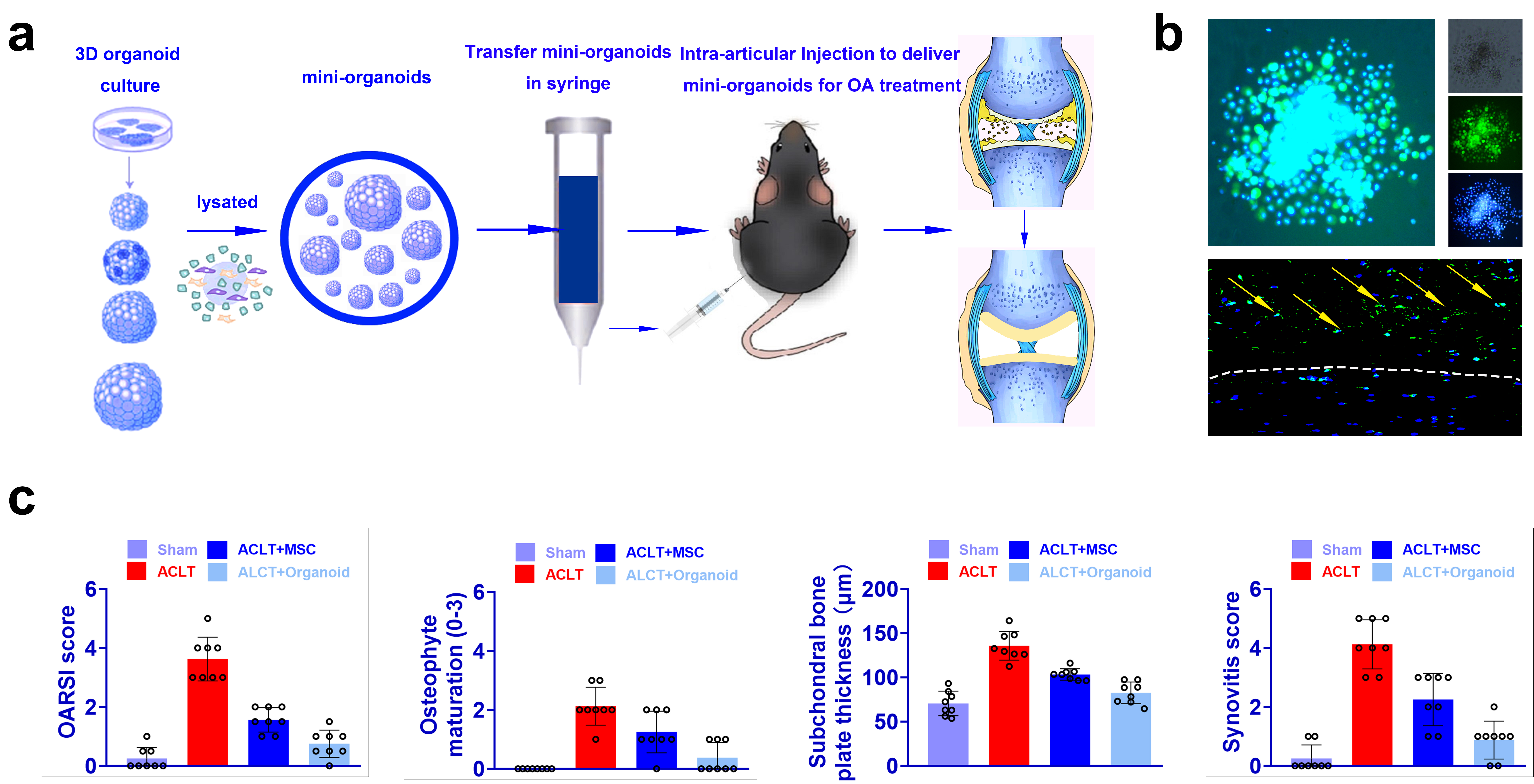

Supplement: Supplementary file 6 — Supplementary Figure 5 [file 41392_2021_675_MOESM6_ESM.jpg]
